# Penicillium marneffei infection in patients with AIDS.

**DOI:** 10.3201/eid0707.017734

**Published:** 2001

**Authors:** T Sirisanthana

**Affiliations:** Chiang Mai University, Chiang Mai, Thailand. ssirisan@med.cmu.ac.th


### *Penicillium marneffei* Infection in Patients with AIDS

**Thira Sirisanthana**
Chiang Mai University, Chiang Mai, Thailand

*Penicillium marneffei* infection (PM) is an important disease among HIV-infected persons in Southeast Asia. Discovered in 1956 from the bamboo rat, *Rhizomys sinensis*, in Vietnam (1), PM was first identified in HIV-infected persons in 1988 (2). The disease has now been reported among HIV-infected persons in Thailand, Myanmar (Burma), Vietnam, Cambodia, Malaysia, northeastern India, Hong Kong, Taiwan, and southern China (3). Cases of PM also have been reported among HIV-infected persons from the United States, the United Kingdom, The Netherlands, Italy, France, Germany, Switzerland, Sweden, Australia, and Japan after they visited the PM-endemic region (3).

PM occurs late in the course of HIV infection. Our study found that the CD4+ cell count at the time of the diagnosis of PM was consistently less than 50 cells/ml. Clinical presentation included fever (in 99% of the patients), anemia (78%), pronounced weight loss (76%), generalized lymphadenopathy (58%), and hepatomegaly (51%). However, these conditions were not specific for PM and could be caused by HIV or other HIV-related opportunistic infections. A more specific finding was skin lesions, most commonly papules with central necrotic umbilication (4), which were seen in 71% of the patients.

In 63% of the patients with PM, a presumptive diagnosis could be made several days before the results of fungal culture were available. This was done by microscopic examination of a Wright-stained sample of bone marrow aspirate, touch smears of a skin biopsy specimen, or a lymph node biopsy specimen. It was easy to culture *P. marneffei* from various clinical specimens. Bone marrow culture was the most sensitive (100%), followed by culture of the specimen obtained from skin biopsy (90%) and blood culture (76%)(4).

The fungus was sensitive to amphotericin B, itraconazole, and ketoconazole (5). The current recommended treatment regimen is to give amphotericin B, 0.6 mg/kg/day for 2 weeks, followed by itraconazole, 400 mg/day orally in two divided doses for the next 10 weeks (6). After initial treatment, the patient should be given itraconazole, 200 mg/day, as secondary prophylaxis for life (7).

Address for correspondence: Thira Sirisanthana, Division of Infectious Diseases, Department of Medicine, Chiang Mai University, Chiang Mai 50200, Thailand; fax: 66-53-217144; e-mail: ssirisan@med.cmu.ac.th

*P. marneffei* has been isolated from several species of bamboo rats in the disease-endemic area, but epidemiologic studies have thus far failed to define an environmental exposure associated with the disease (8-10).

### References

1. Segretain G. Description d'une nouvelle espece de *Penicillium*: *Penicillium marneffei* n. sp. Bull Soc Mycol Fr 1959;75:412-6.
2. Piehl MR, Kaplan RL, Haber MH. Disseminated penicilliosis in a patient with acquired immunodeficiency syndrome. Arch Pathol Lab Med 1988;112:1262-4.
3. Sirisanthana T, Supparatpinyo K. Epidemiology and management of penicilliosis in human immunodeficiency virus-infected patients. Int J Infect Dis 1998;3:48-53.
4. Supparatpinyo K, Khamwan C, Baosoung V, Nelson KE, Sirisanthana T. Disseminated *Penicillium marneffei* infection in Southeast Asia. Lancet 1994;344:110-3.
5. Supparatpinyo K, Nelson KE, Merz WG, Breslin BJ, Cooper CRJr, Kamwan C, et al. Response to antifungal therapy by human immunodeficiency virus-infected patients with disseminated *Penicillium marneffei* infection and in vitro susceptibilities of isolates from clinical specimens. Antimicrob Agents Chemother 1993;37:2407-11.
6. Sirisanthana T, Supparatpinyo K, Perriens J, Nelson KE. Amphotericin B and itraconazole for treatment of disseminated *Penicillium marneffei* infection in human immunodeficiency virus-infected patients. Clin Infect Dis 1998;26:1107-10.
7. Supparatpinyo K, Perriens J, Nelson KE, Sirisanthana T. A controlled trial of itraconazole to prevent relapse of *Penicillium marneffei* infection in patients infected with the human immunodeficiency virus. N Engl J Med 1998;339:1739-43.
8. Chariyalertsak S, Vanittanakom P, Nelson KE, Sirisanthana T, Vanittanakom N. *Rhizomys sumatrensis* and *Cannomys badius*, new natural animal hosts of *Penicillium marneffei*. J Med Vet Mycol 1996;34:105-10.
9. Chariyalertsak S, Sirisanthana T, Supparatpinyo K, Praparattanapan J, Nelson KE. Case-control study of risk factors for *Penicillium marneffei* infection in human immunodeficiency virus-infected patients in northern Thailand. Clin Infect Dis 1997;24:1080-6.
10. Chariyalertsak S, Sirisanthana T, Supparatpinyo K, Nelson KE. Seasonal variation of disseminated *Penicillium marneffei* infection in northern Thailand: a clue to the reservoir? J Infect Dis 1996;173:1490-3.
